# Survival after curative resection for stage I colorectal mucinous adenocarcinoma

**DOI:** 10.1186/s12876-022-02276-z

**Published:** 2022-04-18

**Authors:** Liang Huang, Shuangling Luo, Sicong Lai, Zhanzhen Liu, Huanxin Hu, Mian Chen, Liang Kang

**Affiliations:** Department of Colorectal Surgery, Guangdong Institute of Gastroenterology, and Guangdong Provincial Key Laboratory of Colorectal and Pelvic Floor Diseases, The Sixth Affiliated Hospital of Sun Yat-Sen University, Guangzhou, 510655 Guangdong China

**Keywords:** Mucinous adenocarcinoma, Colorectal carcinoma, Colorectal cancer, Recurrence-free survival, Overall survival

## Abstract

**Purpose:**

The prognostic value of the mucinous adenocarcinoma histotype on the early stages especially for stage I colorectal cancer (CRC) is still unclear. This study determined the clinicopathologic characteristics and long-term outcome of stage I colorectal mucinous adenocarcinomas (MAC).

**Methods:**

Among the total of 530 patients with stage I CRC (58 having MAC and 472 having non-MAC) who underwent radical resection, the correlation between clinicopathological factors and MAC was analyzed. Multivariate analysis was performed to determine whether mucinous histotype itself was an independent prognostic impact in stage I patients.

**Results:**

MACs were observed more frequently located in the colon than rectum (*p* = 0.049), more frequently displayed the deficient mismatch repair (dMMR) phenotype (*p* = 0.001) and had a greater frequency of T2 stage (*p* = 0.002). The rate of recurrence was 15.3% and the mortality was 9.2% among all stage I CRC patients. There was no difference in disease-free survival and overall survival between MACs and non-MACs. On multivariate analysis, older age (*p* = 0.009, hazard ratio: 2.22), rectal cancer (*p* = 0.008, hazard ratio: 3.21), lymphovascular invasion (LVI) (*p* < 0.001, hazard ratio: 6.28), and deficient mismatch repair (dMMR) phenotypes (*p* = 0.044, hazard ratio: 2.62) were independently associated to poor survival of stage I CRC. A high carcinoembryonic antigen level (*p* = 0.034, hazard ratio: 1.86), rectal cancer (*p* = 0.035, hazard ratio: 1.81), LVI (*p* = 0.002, hazard ratio: 3.59) and dMMR phenotypes (*p* = 0.009, hazard ratio: 2.85) were independently related to short disease-free survival of stage I CRC.

**Conclusions:**

Compared with non-MAC, MAC patients had more T2 patients and more dMMR phenotypes in stage I CRC at presentation, but the mucinous histology is not a significant predictor of recurrence and prognosis in stage I CRC.

## Background

Colorectal cancer (CRC) can be classified by histological evaluation of tumor specimens [1, 2], Colorectal mucinous adenocarcinomas (MAC) were defined when the tumor mass consisted 50% or more of mucinous ingredient, mostly extracellular; while the other tumors were defined as non-mucinous adenocarcinomas (non-MAC). Non-MAC is the most common type of CRC (> 85%), while 10–15% of CRC patients are MAC [3]. MAC differs from non-MAC for its special clinicopathological characteristics, compared with non-MAC, MAC has long been associated with an inferior response to treatment, especially radiotherapy and chemotherapy [4]. The debate on the prognostic value of MAC in patients is ongoing, MAC is still considered to be a poor prognosis and refractory subtype of the disease. MAC presents a deficient mismatch repair (dMMR) status, young age and advanced stage at presentation [5, 6]. But the prognostic impact of MAC is controversial, some studies shown that mucinous histology was an independent negative prognostic factor [7, 8], but not in others [9, 10].

In general, MAC patients present a more advanced stage than non-MAC as shown in previous studies [11, 12]. It is well known that the inferior prognostic impact of MAC can be close related to the more advanced progression at presentation [13]. However, most previous studies focused on its clinicopathological characteristics and prognosis of stage III and IV diseases. Moreover, the mucinous pathological subtype can also be a negative factor even for stage II CRC patients [14]. Few studies have focused the impact in survival between MAC and non-MAC of stage I CRC.

Thus, our study aimed to clarify the prognostic impact of MAC focusing on stage I CRC and correlate the mucinous histology with clinicopathological features of stage I CRC.

## Methods

The study protocol was reviewed and approved by the institutional review board of of the Sixth Affiliated Hospital, Sun Yat-sen University, China. The data of all patients are kept confidential and the process of informed consent is abandoned because this study is a retrospective study. This study was carried out in accordance with the recommendations of the Declaration of Helsinki for biomedical research involving human subjects.

### Patient

The study is a single center study, 5753 patients was analysed retrospective, all of them have undergone radical resections due to CRC by specialist surgern in our hospital between January 2011 to May 2016. The following exclusion criteria were applied: patients with familial adenomatous polyposis (FAP), hereditary non-polyposis CRC (HNPCC); patients with synchronous or metachronous cancer; death due to non-cancer causes such as heart disease and cerebral infarction; patients underwent local excision or neoadjuvant therapy were also excluded. Among them, 542 patients (9.42%) were diagnosed as stage I CRC on histopathologic examination and met the criteria for enrollment. Of the 542 patients, 12 patients lost to follow-up. Therefore, 530 patients were analyzed in this study.

### Clinicopathologic evaluation

Before surgery, patients underwent a baseline assessment of demographics and disease characteristics, blood carcinoembryonic antigen (CEA) tests, and tumor imaging. At least two pathologists, who are specialized in CRC, assessed the surgical specimens. Among the 530 patients, 58 were defined as MAC when the tumor mass consisted 50% or more of mucin ingredient, mostly extracellular; and the other tumors were defined as non-MAC. Hematoxylin and eosin staining was used to assess lymph nodes metastasis and lymphovascular invasion (LVI).Immunohistochemically (IHC) assessment of hMLH1, hMSH2, hMSH6, and hPMS2 expression was performed on FFPE samples, as previously described [15]. The clinicopathological features of all 530 patients are shown in Table 1. All patients were staged by TNM classification criteria [16].

**Table 1:** The relationship between clinicopathological characteristics and mucinous adenocarcinoma in stage I colorectal cancer

Variable	MAC (n = 58)	Non-MAC (n = 472)	*p* value
Sex			
Male	34 (58.6%)	255 (54.0%)	0.508
Female	24 (41.4%)	217 (46.0%)	
Age			
≤ 65	40 (69.0%)	291 (61.7%)	0.278
> 65	18 (31.0%)	181 (38.3%)	
Preoperative CEA			
≤ 5 ng/dL	53 (91.4%)	413 (87.5%)	0.393
> 5 ng/dL	5 (8.6%)	59 (12.5%)	
T classification			
1	8 (13.8%)	162 (34.3%)	0.002*
2	50 (86.2%)	310 (65.7%)	
Location			
Colon	24 (41.4%)	136 (28.8%)	0.049*
Rectal	34 (58.6%)	336 (71.2%)	
Size			
< 3 cm	28 (48.3%)	281 (59.5%)	0.101
≥ 3 cm	30 (51.7%)	191 (40.5%)	
LVI			
(−)	54 (93.1%)	457 (96.8%)	0.151
(+)	4 (6.9%)	15 (3.2%)	
Less than 12 lymph nodes			
Yes	10 (17.2%)	87 (18.4%)	0.825
No	48 (82.8%)	385 (81.6%)	
dMMR			
Yes	7 (12.1%)	14 (3.0%)	0.001*
No	51 (87.9%)	458 (97.0%)	

The bold and *  indicates *p* < 0.05

*MAC* mucinous adenocarcinomas, *CEA* carcinoembryonic antigen, *dMMR* deficient mismatch repair

### Treatment

All patients underwent radical surgery. Colon cancer is completely removed by mesocolic excision with Lymph node (LN) dissection at R0-resection level. Resection of rectal cancer were performed by total mesorectal excision as described [17]. Recurrence occurred in 81 of 503 patients during follow-up and 25 patients underwent re-radical surgery. Laparoscopic surgery is performed for most patients.

### Data collection

The follow-up information of 530 patients was collect and analyzed. The median follow-up period of all cases was 45 months (2–103 months). According to the mucinous histology, patients were divided into two groups: the MAC group and the non-MAC group. Clinicopathologic factors (age, sex, preoperative CEA lever, tumor location, tumor size, T stage, histologic subtype, the number of obtained lymph nodes, LVI, MMR status) were analyzed. We selected the 5-year disease-free survival (DFS) as the primary endpoint, which defined as the time from the date of radical resection to the diagnosis of cancer recurrence. The 5-year overall survival rate (OS) was selected as the second endpoint, defined as the time from the date of radical resection to death caused by cancer.

### Statistical analysis

The associations between the discrete variables were analyzed by Spearman rank correlation test. *p* value < 0.05 was regarded as statistically significant. Univariate analyses were performed by χ^2^ tests to evaluate the associations between clinical variables and the tumor histology. The survival probability was analyzed by Kaplan–Meier procedure, and the distribution differences were assessed by the log-rank test. Clinicopathologic factors such as age, sex, preoperative CEA lever, tumor location, tumor size, T stage, histologic subtype, the number of obtained lymph nodes, LVI, MMR status were analyzed. In multivariate analysis, cox proportional risk model (HR) was also used to evaluate the predictive value of various factors. The statistical analysis was carried out by using IBM SPSS ver. 20.0(IBM, Armonk, NY, USA).

## Results

**Fig. 1:**
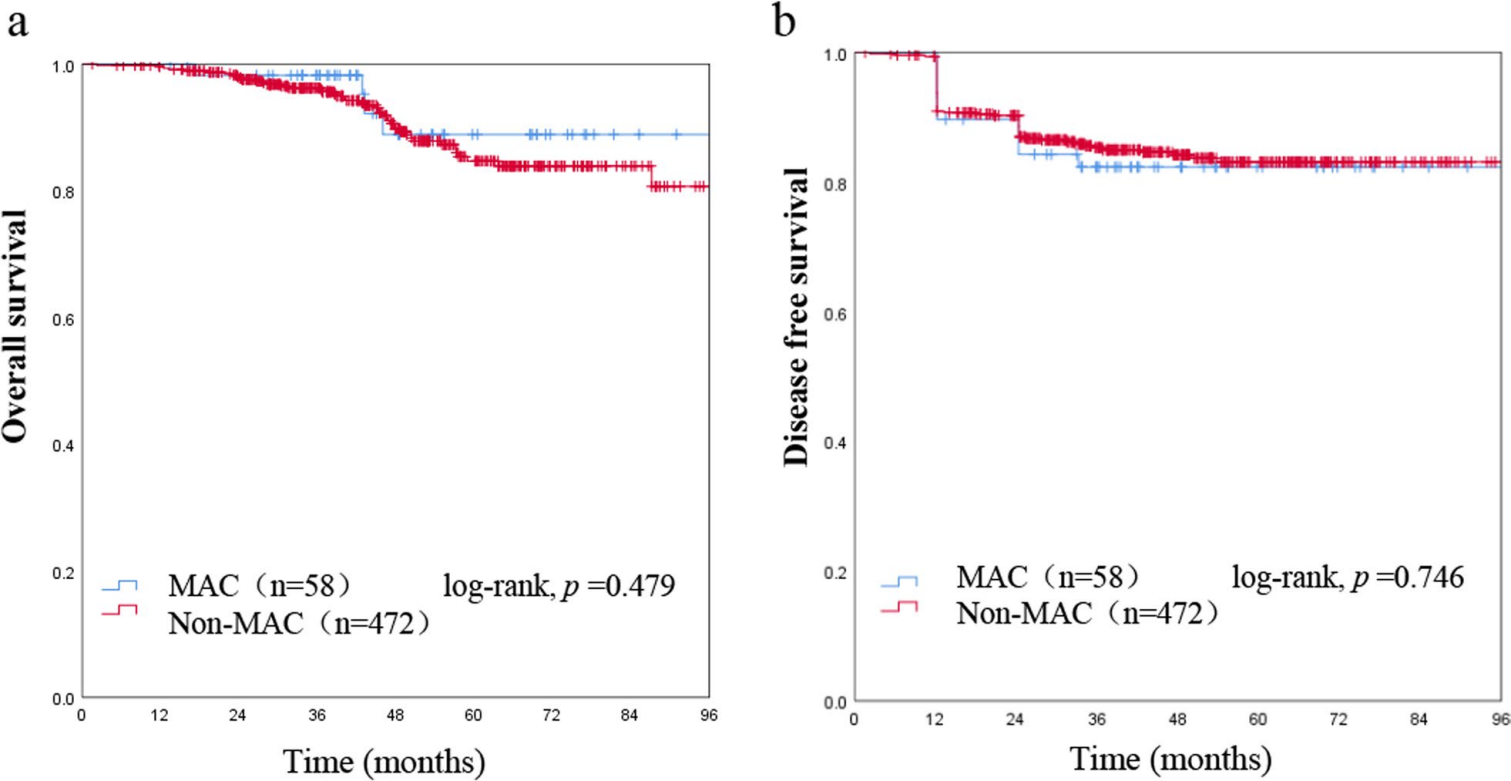
Univariate and multivariate survival analyses, which showed no significant differences in patient survival between mucinous adenocarcinomas (MAC) and non-MAC colorectal cancer, overall survival (**a**) and disease free survival (**b**)

**Fig. 2:**
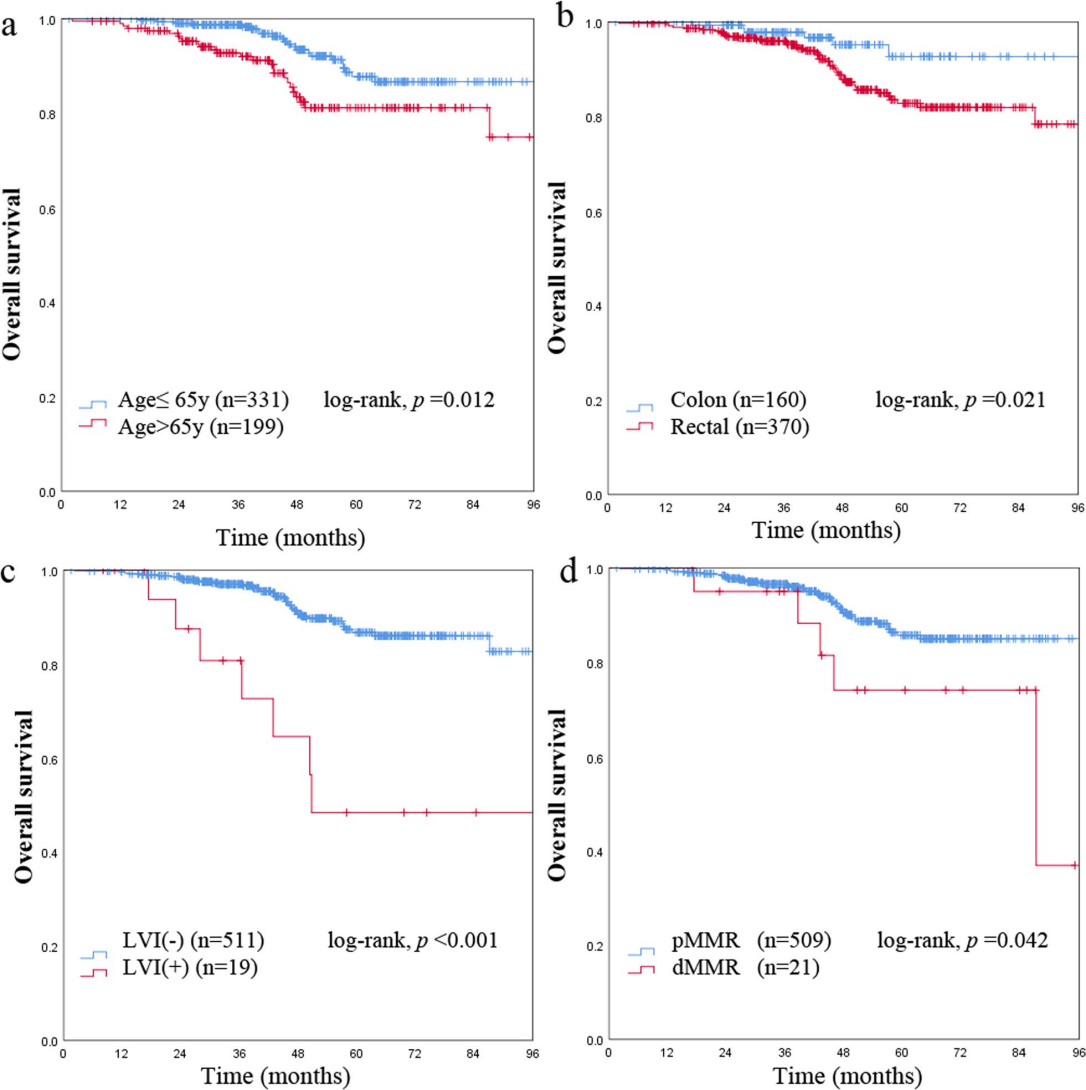
Kaplan–Meier survival curves show that patients being older (**a**), with rectal cancer (**b**), lymphovascular invasion (LVI) positive (**c**), and proficient mismatch repair (pMMR) status (**d**) are significantly related to a poorer overall survival

Table 1 summarized the clinicopathologic characteristics, all of the 530 stage I CRC patients were classified as the MAC group and non-MAC group. MAC was identified in 58 (10.9%) patients by pathology. MAC was found in 34 males (58.6%) and 24 females (41.4%); non-MAC was found in 255 males (54.0%) and 217 females (46.0%). The mean ages of patients with MAC and non-MAC were 57.7 ± 12.6 years and 59.8 ± 12.2 years, respectively (*p* = 0.968). 50 (86.2%) of the 58 patients with MAC had tumors classified as T2 and only 8 (13.8%) as T1. In contrast, 310 (65.7%) of the 472 non-MAC were classified as T2 (*p* = 0.002). The MAC was found to be more frequently locate at colon than the non-MAC (*p* = 0.049). DMMR status was found in 7 (12.1%) patients with MAC and 14 (3.0%) with non-MAC (*p* = 0.001). LVI tended to be more frequent in MAC patients than in non-MAC patients (6.9% vs. 3.2% percent; *p* = 0.151). During the following period, 22 of all stage I patients (530) died of cancer, 27 patients died from non-cancer causes. Relationships between clinicopathological factors and overall survival in all stage I CRC are shown in Table 2. On univariate and multivariate analysis, patients with rectal cancer, being older, LVI positive, dMMR status were found to relate to poorer cancer specific overall survival significantly, however, mucinous histology itself had no significant prognostic effect on OS (Table 2, Figs. 1a and 2). Less than 12 lymph nodes is a risk factor, which may lead to low staging. We divided our patients into two groups, there was no difference in survival between LN < 12 or LN ≥ 12.

**Table 2:** Univariate and multivariate analyses for overall survival in stage I colorectal cancer

Variable	Univariate analysis		Multivariate analysis	
	Mean OS (95% CI)	*p* value	HR (95% CI)	*p* value
Gender				
Male	1	0.604		
Female	0.861 (0.489–1.517)			
Age				
≤ 65	1	0.012*	2.217 (1.207–3.751)	0.009*
> 65	2.030 (1.157–3.563)			
Preoperative CEA				
≤ 5	1	0.250		
> 5	1.596 (0.715–3.566)			
T classification				
T 1	1	0.165		
T 2	1.606 (0.818–3.152)			
Lesion location				
Colon	1	0.021*	3.213 (1.355–7.618)	0.008*
Rectal	2.628 (1.118–6.180)			
Size				
< 3 cm	1	0.884		
≥ 3 cm	0.959 (0.544–1.689)			
Mucinous histology				
MAC	1	0.479		
Non-MAC	1.444 (0.519–4.016)			
LVI				
(−)	1	< 0.001*	6.283 (2.749–14.357)	< 0.001*
(+)	4.666 (2.080–10.465)			
Less than 12 lymph nodes				
Yes	1	0.935		
No	1.031 (0.499–2.130)			
dMMR				
Positive	1	0.042*	2.618 (1.025–6.685)	0.044*
Negative	0.395 (0.156–0.998)			

The bold and *  indicates *p* < 0.05

*MAC* mucinous adenocarcinomas, *CEA* carcinoembryonic antigen, *dMMR* deficient mismatch repair

**Fig. 3:**
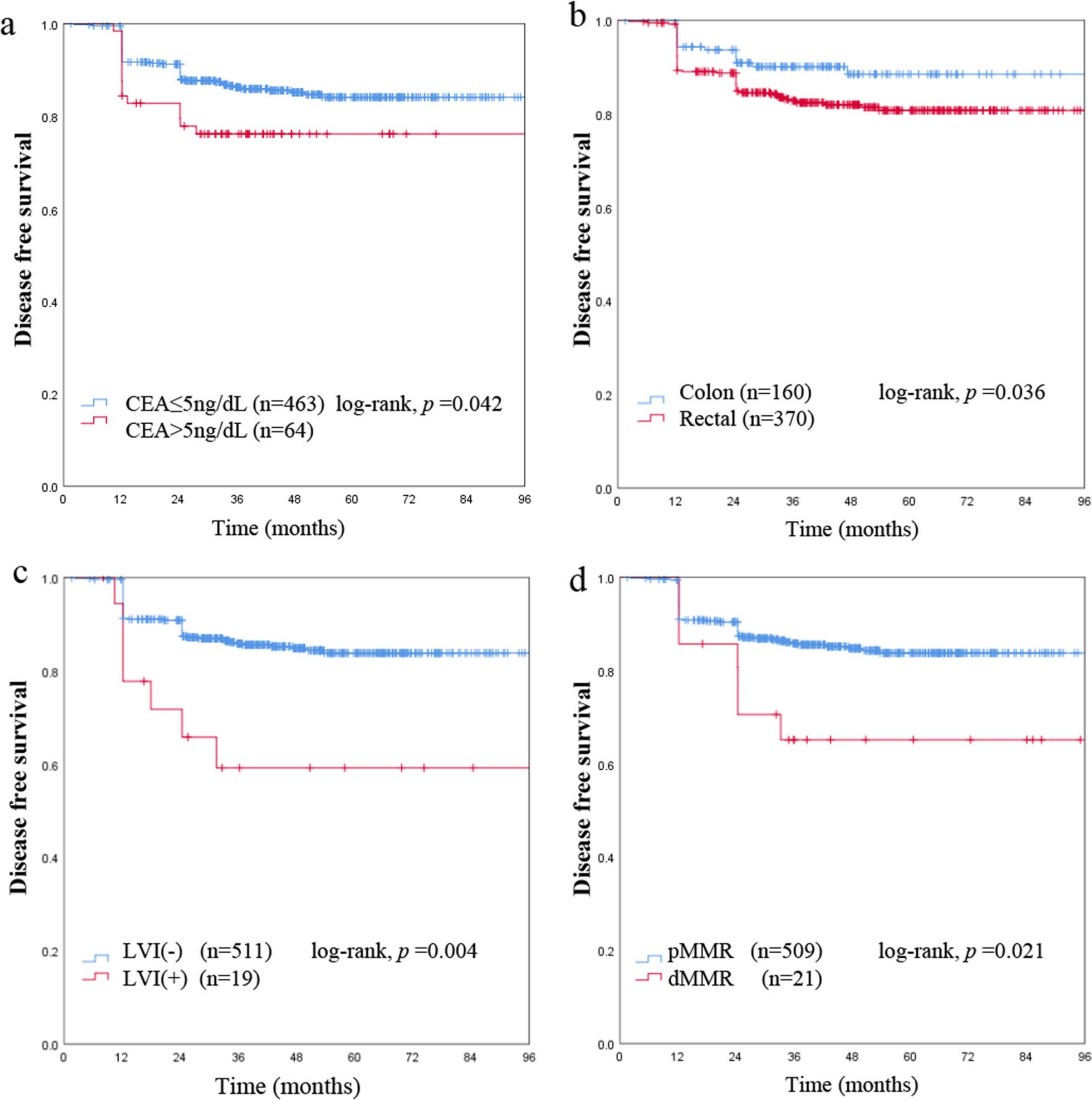
Kaplan–Meier survival curves show that patients with a higher carcinoembryonic antigen (CEA) lever (**a**), rectal cancer (**b**), LVI positive (**c**), and proficient mismatch repair (pMMR) status (**d**) are independently related to disease free survival

81 (15.3%) of the 530 stage I patients experienced recurrence, including 26 (4.9%) with local recurrence and 55 (10.4%) with distant metastasis. Peritoneal dissemination was found in 1 patients with MAC and 4 with non-MAC (*p* = 0.210). Metastasis to the liver was found in 5 patients with MAC and 29 patients with non-MAC (*p* = 0.392). Metastasis to the lung was found in 2 patients with MAC and 15 patients with non-MAC (*p* = 0.524). Most recurrences occurred within 2 years after operation, as shown in Fig. 1b. On univariate and multivariate survival analysis, patients with rectal cancer, higher CEA lever, LVI positive, dMMR status were independently related to short DFS, while mucinous histology was also not a significantly predictor for recurrence (Table 3, Figs. 1b and 3).

**Table 3:** Univariate and multivariate analyses for disease free survival in stage I colorectal cancer

Variable	Without recurrence (n = 449)	With recurrence (n = 81)	Univariate *p* value	Multivariate	
				HR (95% CI)	*p* value
Gender					
Male	241 (53.7%)	48 (59.3%)	0.268		
Female	208 (46.3%)	33 (40.7%)			
Age					
≤ 65	287 (63.9%)	44 (54.3%)	0.090		
> 65	162 (36.1%)	37 (45.7%)			
Preoperative CEA					
≤ 5	400 (89.1%)	66 (81.5%)	0.042*	1.863 (1.049–3.310)	0.034*
> 5	49 (10.9%)	15 (18.5%)			
T classification					
T1	149 (33.2%)	21 (25.9%)	0.243		
T2	300 (66.8%)	60 (74.1%)			
Lesion location					
Colon	144 (32.1%)	16 (19.8%)	0.036*	1.813 (1.041–3.156)	0.035*
Rectal	305 (67.9%)	65 (80.2%)			
Size					
< 3 cm	260 (57.9%)	49 (60.5%)	0.527		
≥ 3 cm	189 (42.1%)	32 (39.5%)			
Mucinous histology					
MAC	48 (10.7%)	10 (12.3%)	0.746		
Non-MAC	401 (89.3%)	71 (87.7%)			
LVI					
(−)	437 (97.3%)	74 (91.4%)	0.004*	3.587 (1.627–7.909)	0.002*
(+)	12(2.7%)	7 (8.6%)			
Less than 12 lymph nodes					
Yes	76 (16.9%)	21 (25.9%)	0.583		
No	373 (83.1%)	60 (74.1%)			
dMMR					
Positive	14 (2.7%)	7 (8.6%)	0.021*	2.848 (1.300–6.238)	0.009*
Negative	435 (97.3%)	74 (91.4%)			

The bold and *  indicates *p* < 0.05

*MAC* mucinous adenocarcinomas, *CEA* carcinoembryonic antigen, *dMMR* deficient mismatch repair

## Discussion

It is difficult to determine the clinicopathologic characteristics and long-term outcome of stage I MAC. Even though the long-term survival of stage I CRC is thought to be much better, it is still unclear whether mucinous histology had significant prognostic effect on stage I CRC. Previous research shows poor survival for CRC patients with a higher mucinous content [18, 19], but there were also reports with opposite conclusions [20, 21]. Here, we analyzed the prognostic value of MAC focusing on stage I CRC and correlated the mucinous histology with clinical and pathological features of stage I CRC. Compared with non-MAC patients, MAC had more T2 patients in stage I at presentation, more colon cancers and a higher dMMR status. Many independent risk factors for recurrence and long-term survival were found in our study, including abnormal CEA level, LVI and dMMR phenotypes. However, there was no correlation between mucinous histology and survival in stage I CRC.

In our study, univariate and multivariate analyses showed that that the histology pattern of MAC was not a prognostic factor for DFS or OS. Although the pathological T2-classification of MAC was higher in stage I patients than in non-MAC patients (86.2% vs. 65.7%, *p* = 0.002), distinct clinical results were not observed. Du et al. [22] also reported that patients with MAC in stage III alone shown poorer DFS and OS compared with the non-MAC group, which means the worse survival of MAC patients might be due to regional lymph node metastasis.

Serum CEA is the most widely used tumor markers for diagnosis and recurrence monitoring of CRC. Several studies have investigated the ability of CEA to predict tumor recurrence and metastasis [23–25]. However, in stage I CRCs, there has been a paucity of evidence for it being a predictive factor for recurrence. Here, we found abnormal pretreatment CEA was an independent risk factor for recurrence even in stage I CRC. It suggests that if the serum CEA level is high preoperative in patients, the recurrence of CRC should be closely monitored, even in stage I CRC.

LVI is considered to be an early event in lymph node metastasis, and it has been proved to be an independent predictor of survival in CRC. The LVI group showed a higher risk of recurrence and a significantly lower overall survival rate in advanced CRC compared with the non-LVI group [26]. Meanwhile, LVI also has an independent predictor power for poor prognosis rectal cancer after neoadjuvant therapy and surgery [27]. However, the prognostic value of LVI in stage I CRC patient has not been well studied. Our study confirms that LVI is an important risk factor for stage I CRC recurrence. This suggests that LVI might be a sensitive marker for local recurrence and distant metastasis, even the patients without LN metastases.

The effect of dMMR on the prognosis of CRC is controversial. DMMR status influences the prognosis of CRC only in specific stages [28, 29]. Compared to pMMR patients, dMMR patients were found to possess worse survival in stage III colon cancer [30]. While opposite conclusion was found that dMMR status was related to a better survival in stage II CRC [31, 32]. Our study revealed that stage I CRC patients with dMMR status showed a worse survival compared with the pMMR group, The result was contradicted with the previous reports, and this may be that patients with mucinous adenocarcinoma are not sensitive to chemotherapy and immunotherapy once they have recurrence and metastasis [33]. In addition, there were only 21 patients with dMMR in our study, and the small sample size may also affect the results. Therefore, we will further expand the sample size or conduct multi-center research in the future.

## Conclusions

This study shows that compared with non-MAC, MAC patients had more dMMR phenotypes and T2 patients in stage I CRC at presentation, but mucinous histology is not an important predictor of recurrence and prognosis in stage I CRC.

## Data Availability

The datasets used and/or analyzed during the current study are available from the corresponding author on reasonable request.
